# An Adaptive Channel Access Method for Dynamic Super Dense Wireless Sensor Networks

**DOI:** 10.3390/s151229800

**Published:** 2015-12-03

**Authors:** Chunyang Lei, Hongxia Bie, Gengfa Fang, Xuekun Zhang

**Affiliations:** 1 School of Information and Communication Engineering, Beijing University of Posts and Telecommunications, Beijing 100876, China; biehx@bupt.edu.cn (H.B.); zhangxuekun1990@gmail.com (X.Z.); 2 Department of Engineering, Macquarie University, Sydney 2109, Australia; gengfa.fang@mq.edu.au

**Keywords:** backoff algorithm, MAC Protocol, Wireless Sensor Network

## Abstract

Super dense and distributed wireless sensor networks have become very popular with the development of small cell technology, Internet of Things (IoT), Machine-to-Machine (M2M) communications, Vehicular-to-Vehicular (V2V) communications and public safety networks. While densely deployed wireless networks provide one of the most important and sustainable solutions to improve the accuracy of sensing and spectral efficiency, a new channel access scheme needs to be designed to solve the channel congestion problem introduced by the high dynamics of competing nodes accessing the channel simultaneously. In this paper, we firstly analyzed the channel contention problem using a novel normalized channel contention analysis model which provides information on how to tune the contention window according to the state of channel contention. We then proposed an adaptive channel contention window tuning algorithm in which the contention window tuning rate is set dynamically based on the estimated channel contention level. Simulation results show that our proposed adaptive channel access algorithm based on fast contention window tuning can achieve more than 95% of the theoretical optimal throughput and 0.97 of fairness index especially in dynamic and dense networks.

## 1. Introduction

Distributed Wireless Sensor Networks (WSNs) play an important role of monitoring and sensing wide-range of environmental parameters in the current and future surveillance systems. Thanks to the tremendous range of applications that they can enable, distributed WSNs become an essential part of 5G networks where Internet of Things (IoT), Machine-to-Machine (M2M) and Vehicular-to-Vehicular (V2V) networks are the promising new applications. As a result, distributed WSNs will become extremely dense in the future. Highly dense wireless sensors need to be deployed in order to sense environmental parameters as a part of the smart cities [1,2]. Highly dense development of sensors is also essential to support applications, such as the high precise seismic exploration and pollution monitoring [3,4], where there are up to thousands of wireless sensors accessing the channel dynamically within an area of 1000 square meters.

How to design such highly dense and distributed wireless sensor network and more specifically the channel access algorithm to support extremely large contending nodes accessing the channel are new challenges. While the wireless sensors have become much denser, contention of accessing wireless channels among neighboring nodes has increased exponentially and become very dynamic depending on whether the neighboring nodes have data for transmission or not. The number of contending nodes can vary from tens to hundreds. Furthermore, the number of contending nodes changes more quickly and frequently than the classic sensor networks. For example, a sudden change of contending nodes from several to several hundreds may happen when there is an event happening in an area where all the nodes have data to report. In super dense WSNs, new adaptive and efficient channel access control protocol and algorithm are required to achieve efficient wireless channel access by avoiding high congestion among the nodes.

In WSNs, contention based carrier sense multiple access with collision avoidance (CSMA/CA) protocol is applied to enable the channel accessing by multiple nodes in a distributed way. According to CSMA/CA, WSN nodes generate random backoff time based on the predefined contention window size before accessing the channel. A well designed scheme to control the contention window size based on the contending nodes nearby can efficiently solve the channel contention problem [5]. In this paper, we focus on analyzing CSMA/CA based channel access and contention problem, and proposing a new channel accessing algorithm which is adaptive to the dynamics of the contending nodes.

Binary Exponential Backoff (BEB) [6,7] has been widely used as the channel access protocol for WSNs based on IEEE 802.15.4 Zigbee [8] or IEEE 802.11x WiFi [9,10]. By increasing the contention window size exponentially after each successful channel access, BEB tries to schedule the nodes accessing the channel in a distributed and efficient way. However, its performance decreases rapidly when the number of contending nodes becomes very large. Previous work in [11] indicates that more than 40% throughput is lost when the number of contending nodes reaches 60 for Zigbee/WiFi based sensor networks. Others [12,13,14,15] proposed new backoff algorithms such as EIED and MILD based on BEB in which contention window is tuned exponentially or linearly after each failure of channel access attempt. The above proposed schemes have poor overall network performance in dense networks for lack of the knowledge of channel congestion level when the number of contending nodes becomes large.

Authors in [16,17] analyzed the relationship between different number of contending nodes and their corresponding optimal contention window size, based on which a series of contention based backoff algorithms were proposed in [18,19,20]. These algorithms firstly try to estimate the number of nodes trying to access the channel simultaneously by continuously monitoring the channel state, and then tune the contention window accordingly. It is not easy to estimate the exact number of contending nodes on-the-fly especially in the super dense and dynamic networks where the number of contending nodes changes dramatically from time to time. What is more, an accurate estimation always takes longer time and ultra higher computation complexity, which is not affordable for low power and low computing power nodes in WSNs.

Considering the problems above, the authors in [21] proved that the probability of an idle state keeps constant when the network throughput approaches the theoretical upper bound of the channel. As a result, lots of idle state sensing based algorithms were proposed [22,23,24]. Without estimating the exact number of contending nodes, the proposed algorithms try to tune the contention window according to whether the estimated channel state parameter is optimal or not. These methods can effectively improve the network throughput when the number of contending nodes is very large. However, the contention window tuning speed is not fast enough to cope with a situation where there is a fast and dramatic change of the contending nodes. What is more, as the estimation results are obtained based on a limited number of samples, large estimation error is inevitable. In idle state sensing based algorithms, the contention window tuning rate is limited to small value only, otherwise the contention window size will not be able to reach its optimum because of the inaccurate estimation. A small tuning rate takes much longer period of time before the contention window can reach its optimal. If the number of contending nodes changes faster than the contention window tuning rate, network throughput will decline severely because the contention window is not always at its optimal size. Considering the issues above, the following parameters are used in this paper to evaluate the performance of backoff algorithms:

- **Tuning Accuracy:** defines how well the contention window of the backoff algorithm fits the channel contention based on the contention levels and number of contending nodes.
- **Tuning Speed:** defines how fast the backoff algorithm can tune the contention window to the optimal size whenever there is a change of the number of contending nodes.

In this paper, a normalized channel contention analysis model is proposed considering the property of dynamic channel contention. In this model, we introduce a series of transformations to map channel conditions onto pre-defined reference cases. For each reference case, we can easily calculate accurate indications about the contention window and its tuning strategy. We also prove the accuracy of the model. In order to speed up the contention window tuning process without sacrificing the tuning accuracy, we propose a contention window tuning scheme through adaptive tuning rate. In this method, the tuning rate is calculated based on the gap between the current contention window size and the channel contention level. When the gap is big, a large tuning step is applied to make sure the contention window can quickly reach to its optimal to improve the turning speed, otherwise a small tuning step is chosen to improve the tuning accuracy.

The rest of this paper is organized as follows. In Section 2 we setup and verify a normalized channel contention analysis model. An adaptive contention window tuning scheme is proposed and studied in Section 3. In Section 4, we study the performance of the proposed algorithm both in static and dynamic settings of networks through simulations. In Section 5 we conclude this paper.

## 2. Normalized Channel Contention Analysis

According to the CSMA/CA protocol, a contending node can not access the channel directly by sending data even when it senses a free channel. Instead, it will firstly need to wait for a random interval decided by the contention window before it starts accessing the channel which is the key part of the backoff algorithms. Channel contention analysis plays a key role in the design of backoff algorithms since it helps to understand how to do the tuning of contention window and its impact on the channel access in terms of throughput and fairness is huge. As specified by the previous research, channel contention level indicates the average number of contending nodes which try to send data through the wireless channel simultaneously in a given time slot (time slot: the unit time in Zigbee/WiFi networks [9]). Therefore, the channel contention level can be represented by the number of nodes that are contending to access the channel and their corresponding probability of accessing the channel successfully in a given slot. By modeling the backoff process as a Markov chain, authors in [25] defined the probability of successfully accessing the channel in the current slot as below Equation (1),

(1) $$\tau =\frac{2}{cw+1}$$

where cw is a node’s contention window size. The contention level then can be defined as a function of the number of contending nodes *n* and the contention window size cw. We denote the contention level as C(cw,n). (The detailed definition of the function C(cw,n) will be given in next subsection.)

We divide the process of backoff into two steps: the first step is about estimating the contention level in current channel indicated by C(cw,n), and the second step is to calculate the contention window so as to optimize the contention level where we model the selection of the contention window as a matching process problem between cw, *n* and C(cw,n). Previous research [26] focuses on more ideal settings where *n* and cw are perfectly matched without considering cases in highly dense networks where C(cw,n) is far more complicated. The mismatched cases are very important in determining the contention window tuning proceeding in highly dense networks in practice.

### 2.1. Model Construction

In order to deal with the mismatched cases, we introduce a correlation coefficient *θ* and define it as θ=n/cw. Thus we have C(cw,n)=C(cw,θ·cw). We define $C_{1}\left(cw,\theta \right)=C\left(cw,\theta \cdot cw\right)$. The mappings between $C_{1}\left(cw,\theta \right)$ and C(cw,n) are presented in Equation (2), where *θ* represents a matching degree between the contention window size and the channel contention state decided by the number of contending nodes. In Equation (2), *a* is an arbitrary positive number, which indicates the linear variety of cw and *θ*.

(2) $$\left\{\begin{array}{c} C_{1}\left(cw,a\cdot \theta \right)=C\left(cw,a\cdot \theta \cdot cw\right)=C\left(cw,a\cdot n\right)\\ C_{1}\left(a\cdot cw,\theta \right)=C\left(a\cdot cw,\theta \cdot \left(a\cdot cw\right)\right)=C\left(a\cdot cw,a\cdot n\right) \end{array}\right.$$

By combining the function of cw and *θ* in $C_{1}\left(cw,\theta \right)$, we can notice that although channel contention level changes as a function of *θ*, it may remain static if cw and *n* are increased and decreased accordingly. We introduce a constant contention window size $cw_{ref}$, so that $C_{1}\left(cw,\theta \right)$ can be expressed as follows Equation (3),

(3) $$C_{1}\left(cw,\theta \right)=C_{1}\left(cw_{ref},\theta \right)+N\left(cw\right)$$

In Equation (3), N(cw) was defined as the difference between $C_{1}\left(cw,\theta \right)$ and $C_{1}\left(cw_{ref},\theta \right)$. If we can prove that N(cw) is small enough compared to the value of $C_{1}\left(cw_{ref},\theta \right)$, $C_{1}$ can be further simplified as a function of one variable plus the noise. Moreover, if the noise is small enough, the expression of $C_{1}$ can be further simplified. We denote the simplified $C_{1}\left(cw,\theta \right)$ as $C_{2}\left(\theta \right)$. Mapping C(cw,n) onto $C_{2}\left(\theta \right)$ brings significant difference to the design of backoff algorithms.

Although the expression of C(cw,n) has been simplified, original C(cw,n) is still embedded in $C_{2}\left(\theta \right)$ which is important to improve the backoff algorithm. If the reference cases are properly designed, we can significantly simplify the algorithm. The change of channel contention level will be indicated by a deviation of *θ*. By utilizing its deviation degree, the contention window can be tuned more efficiently in terms of throughput and speed. As some of the cases with different cw have the same *θ*, we can dramatically reduce the complexity of the proposed algorithm which is important in practice.

### 2.2. Model Verification

The validity of the proposed model can be evaluated through |N(cw)| which is the absolute deviation of the mapping as in Equation (3). The upper bound of |N(cw)| can be calculated as Equation (4)

(4) $$\begin{array}{cc} \left|N(cw)\right| & =\;|C_{1}\left(cw,\theta \right)-C_{1}\left(cw_{ref},\theta \right)|\\ & =\;|\int_{cw_{ref}}^{cw}\frac{\partial C_{1}\left(t,\theta \right)}{\partial t}dt|\leq |cw-cw_{ref}\left|\cdot max\{\right|\frac{\partial C_{1}\left(t,\theta \right)}{\partial t}\left|,t\in [cw_{ref},cw]\right\} \end{array}$$

According to work in [11,27,28], the contention of accessing the channel can be modeled as a discrete time stochastic process that includes three states: idle slot state, successful packet transmission state including RTS/CTS/DATA/ACK and failed transmission state indicated by a failed RTS. The probabilities of the above states are three basic parameters in CSMA/CA based channel access schemes. They can be used to indicate the contention level in channel. Other channel parameters can be calculated based on the three parameters above. Thus evaluating |N(cw)| based on $C_{1}\left(cw,\theta \right)$ equals to evaluating |N(cw)| based on the three basic states.

Next we will focus on calculating the upper bound of |N(cw)| for the three cases where channel contention level is determined by the probability of idle slot state, the probability of successful transmission state, and the probability of failed transmission state accordingly.

#### 2.2.1. Case 1: Channel Contention Level Depends on the Probability of Idle Slot State

An idle slot state will appear if there is no node having data for transmission. Thus the probability of an idle slot state can be expressed as Equation (5) below.

(5) $$P_{Idle}\left(n,\tau \right)={\left(1-\tau \right)}^{n}$$

By substituting n=θ·cw and Equation (1) to Equation (5), the probability of an idle channel becomes:

(6) $$P_{I}\left(cw,\theta \right)={\left(\frac{cw-1}{cw+1}\right)}^{\theta \cdot cw}$$

We can have the first-order partial derivative of $P_{I}\left(cw,\theta \right)$:

(7) $$\frac{\partial P_{I}\left(cw,\theta \right)}{\partial cw}=P_{I}\left(cw,\theta \right)\cdot \left(\theta \cdot \ln\frac{cw-1}{cw+1}+\frac{2\theta \cdot cw}{cw^{2}-1}\right)$$

In order to estimate $\partial P_{I}\left(cw,\theta \right)/\partial cw$, we continue to calculate the second-order partial derivative of $P_{I}\left(cw,\theta \right)$.

Let

(8) $$X\left(cw,\theta \right)=\theta \cdot \ln\frac{cw-1}{cw+1}+\frac{2\theta \cdot cw}{cw^{2}-1}$$

and

(9) $$\frac{\partial X(cw,\theta )}{\partial cw}=\frac{-4\theta }{{\left(cw^{2}-1\right)}^{2}}$$

Thus

(10) $$\frac{\partial P_{I}\left(cw,\theta \right)}{\partial cw}=P_{I}\left(cw,\theta \right)\cdot X\left(cw,\theta \right)$$

Then we can have

(11) $$\begin{array}{c} \frac{\partial^{2}P_{I}\left(cw,\theta \right)}{\partial cw^{2}}=P_{I}\left(cw,\theta \right)\cdot \left[X{\left(cw,\theta \right)}^{2}+\frac{-4\theta }{{\left(cw^{2}-1\right)}^{2}}\right] \end{array}$$

Obviously, ∂X(cw,θ)/∂cw<0 which means that X(cw,θ) decreases as a function of cw. As $\lim_{cw\to +\infty }X\left(cw,\theta \right)=0$ for every *θ*, we have X(cw,θ)>0. Since $P_{I}\left(cw,\theta \right)\in \left[0,1\right]$, we can have $\partial P_{I}\left(cw,\theta \right)/\partial cw>0$.

We define

(12) $$X_{a}\left(cw,\theta \right)=\sqrt{-\frac{\partial X(cw,\theta )}{\partial cw}}=\frac{2\sqrt{\theta }}{cw^{2}-1}$$

We then have

(13) $$\begin{array}{c} \frac{\partial X_{a}\left(cw,\theta \right)}{\partial cw}=\frac{-4\sqrt{\theta }\cdot cw}{{\left(cw^{2}-1\right)}^{2}} \end{array}$$

Now we can combine Equation (11) with Equation (12) and we can get

(14) $$\begin{array}{c} \frac{\partial^{2}P_{I}\left(cw,\theta \right)}{\partial cw^{2}}=P_{I}\left(cw,\theta \right)\cdot \left[X\left(cw,\theta \right)+X_{a}\left(cw,\theta \right)\right]\cdot \left[X\left(cw,\theta \right)-X_{a}\left(cw,\theta \right)\right] \end{array}$$

From Equations (9) and (13), we can get $\partial [X\left(cw,\theta \right)+X_{a}\left(cw,\theta \right)]/\partial cw<0$ and $\partial [X\left(cw,\theta \right)-X_{a}\left(cw,\theta \right)]/\partial cw>0$. By combining the conditions $\lim_{cw\to +\infty }\left[X\left(cw,\theta \right)\pm X_{a}\left(cw,\theta \right)\right]=0$, we have $X\left(cw,\theta \right)+X_{a}\left(cw,\theta \right)>0$ and $X\left(cw,\theta \right)-X_{a}\left(cw,\theta \right)<0$. For $P_{I}\left(cw,\theta \right)\in \left[0,1\right]$, we can get $\partial^{2}P_{I}\left(cw,\theta \right)/\partial cw^{2}<0$.

From the above we can see that $\partial P_{I}\left(cw,\theta \right)/\partial cw$ monotonously approaches 0 with respect to cw. Therefore, for any $cw>cw_{ref}$ we have

(15) $$\begin{array}{c} \max\{|\frac{\partial P_{I}\left(t,\theta \right)}{\partial t}|,t\in \left[cw_{ref},cw\right]\}\;=\;|\frac{\partial P_{I}\left(cw_{ref},\theta \right)}{\partial cw_{ref}}| \end{array}$$

Thus according to Equation (4), we can have the upper bound of N(cw) in Equation (16) for any $cw>cw_{ref}$ as below,

(16) $$\left|N\left(cw\right)\right|\leq \left(cw-cw_{ref}\right)\cdot \left|\frac{\partial P_{I}\left(cw_{ref},\theta \right)}{\partial cw_{ref}}\right|$$

#### 2.2.2. Case 2: Channel Contention Level in the Successful Transmission State

For this case, the successful transmission in a given slot happens when there is only one node having data for transmission and the rest nodes do not have data. Thus the probability of successful transmission state can be expressed as:

(17) $$P_{success}\left(n,\tau \right)=n\tau {\left(1-\tau \right)}^{n-1}$$

By substituting n=θ·cw and Equation (1) to Equation (17), we can have the next expression:

(18) $$P_{S}\left(cw,\theta \right)=\frac{2\theta \cdot cw}{cw-1}\cdot {\left(\frac{cw-1}{cw+1}\right)}^{\theta \cdot cw}$$

By defining

(19) $$Y\left(cw,\theta \right)=\frac{-1}{cw\cdot (cw-1)}$$

We can have

(20) $$\frac{\partial Y(cw,\theta )}{\partial cw}=\frac{2cw-1}{cw^{2}\cdot {\left(cw-1\right)}^{2}}$$

and then we have

(21) $$\frac{\partial P_{S}\left(cw,\theta \right)}{\partial cw}=P_{S}\left(cw,\theta \right)\cdot \left[X\left(cw,\theta \right)+Y\left(cw,\theta \right)\right]$$

Thus we can have

(22) $$\frac{\partial^{2}P_{S}\left(cw,\theta \right)}{\partial cw^{2}}=P_{S}\left(cw,\theta \right)\cdot \left\{{\left[X\left(cw,\theta \right)+Y\left(cw,\theta \right)\right]}^{2}+\frac{\partial X(cw,\theta )}{\partial cw}+\frac{\partial Y(cw,\theta )}{\partial cw}\right\}$$

It is obvious that ∂X(cw,θ)/∂cw+∂Y(cw,θ)/∂cw>0. Since $P_{S}\left(cw,\theta \right)\in \left[0,1\right]$, we have $\partial^{2}P_{S}\left(cw,\theta \right)/\partial cw^{2}>0$. As ∂X(cw,θ)/∂cw+∂Y(cw,θ)/∂cw>0, and $\lim_{cw\to +\infty }\left[X\left(cw,\theta \right)+Y\left(cw,\theta \right)\right]=0$, we have $\partial P_{S}\left(cw,\theta \right)/\partial cw<0$.

Based on the above, we can conclude that $P_{S}\left(cw,\theta \right)$ monotonously approaches 0 with respect to cw. Therefore, for any $cw>cw_{ref}$, we have

(23) $$\begin{array}{c} \max\{|\frac{\partial P_{S}\left(t,\theta \right)}{\partial t}|,t\in \left[cw_{ref},cw\right]\}\;=\;|\frac{\partial P_{S}\left(cw_{ref},\theta \right)}{\partial cw_{ref}}| \end{array}$$

According to Equation (4), for any $cw>cw_{ref}$ we can get the upper bound of N(cw) according to Equation (24) below,

(24) $$\left|N\left(cw\right)\right|\leq \left(cw-cw_{ref}\right)\cdot \left|\frac{\partial P_{S}\left(cw_{ref},\theta \right)}{\partial cw_{ref}}\right|$$

#### 2.2.3. Case 3: Channel Contention Level in the Conflicting Transmission State

Since the idle slot state, successful packet transmission state and conflicting packet transmission state are the only three possible states while accessing the channel, the probability of conflicting packet transmission state $P_{C}\left(cw,\theta \right)$ and its corresponding first-order partial derivative can be calculated as Equations (25) and (26) as below accordingly:

(25) $$P_{C}\left(cw,\theta \right)=1-P_{I}\left(cw,\theta \right)-P_{S}\left(cw,\theta \right)$$

(26) $$\frac{\partial P_{C}\left(cw,\theta \right)}{\partial cw}=-\frac{\partial P_{I}\left(cw,\theta \right)}{\partial cw}-\frac{\partial P_{S}\left(cw,\theta \right)}{\partial cw}$$

According to Section 2.2.1 and Section 2.2.2, we can have

(27) $$\frac{\partial P_{I}\left(cw,\theta \right)}{\partial cw}\cdot \frac{\partial P_{S}\left(cw,\theta \right)}{\partial cw}<0$$

Therefore, for any $cw<cw_{ref}$ we have

(28) $$\max\{|\frac{\partial P_{C}\left(t,\theta \right)}{\partial t}|,t\in \left[cw_{ref},cw\right]\}\;=\max\{|\frac{\partial P_{S}\left(cw,\theta \right)}{\partial cw}\left|,\right|\frac{\partial P_{I}\left(cw,\theta \right)}{\partial cw}\left|\right\}$$

Thus according to Equation (4), we can calculate the upper bound of N(cw) as in Equation (29) for any $cw>cw_{ref}$.

(29) $$\left|N\left(cw\right)\right|\leq \left(cw-cw_{ref}\right)\cdot \max\{|\frac{\partial P_{I}\left(cw_{ref},\theta \right)}{\partial cw_{ref}}\left|,\right|\frac{\partial P_{S}\left(cw_{ref},\theta \right)}{\partial cw_{ref}}\left|\right\}$$

#### 2.2.4. The Analysis of System Throughput

According to the above analysis of $P_{I}\left(cw,\theta \right)$, $P_{S}\left(cw,\theta \right)$ and $P_{C}\left(cw,\theta \right)$, the system throughput S(cw,θ) can be expressed as Equation (30) [25] as follows,

(30) $$S\left(cw,\theta \right)=\frac{L_{data}\cdot P_{S}\left(cw,\theta \right)}{T_{S}\cdot P_{S}\left(cw,\theta \right)+T_{C}\cdot P_{C}\left(cw,\theta \right)+T_{I}\cdot P_{I}\left(cw,\theta \right)}$$

where $L_{data}$ is the average frame size at the physical layer, and $T_{I}$, $T_{S}$, $T_{C}$ are the average time durations of the three states accordingly which can be estimated by PHY and MAC layers [9].

By separating the variable cw and *θ* and substituting Equation (25) to Equation (30), we have:

(31) $$S_{r}\left(cw,\theta \right)=\frac{L_{data}}{T_{S}-T_{C}+T_{C}\cdot \left[P_{S}^{-1}\left(cw,\theta \right)-\eta \cdot P_{I}\left(cw,\theta \right)\cdot P_{S}^{-1}\left(cw,\theta \right)\right]}\;,\;\;\;\eta =1-\frac{T_{I}}{T_{C}}$$

We then define

(32) $$\Lambda \left(cw,\theta \right)=P_{S}^{-1}\left(cw,\theta \right)-\eta \cdot P_{I}\left(cw,\theta \right)\cdot P_{S}^{-1}\left(cw,\theta \right)$$

According to Equations (6)–(9) and (18)–(20), we can get the first-order and second-order partial derivatives of Λ(cw,θ) as shown in Equations (33) and (34) below.

(33) $$\frac{\partial \Lambda (cw,\theta )}{\partial cw}=-\frac{1}{P_{S}\left(cw,\theta \right)}\cdot \left(X\left(cw,\theta \right)+Y\left(cw,\theta \right)-\eta \cdot P_{I}\left(cw,\theta \right)\cdot Y\left(cw,\theta \right)\right)>0$$

(34) $$\begin{array}{cc} \frac{\partial^{2}\Lambda \left(cw,\theta \right)}{\partial cw^{2}}= & \frac{1}{P_{S}\left(cw,\theta \right)}{\cdot [\left(X\left(cw,\theta \right)+Y\left(cw,\theta \right)\right)}^{2}-\left(\frac{\partial X(cw,\theta )}{\partial cw}+\frac{\partial Y(cw,\theta )}{\partial cw}\right)\\ & -\eta \cdot P_{I}\left(cw,\theta \right)\cdot \left(Y{\left(cw,\theta \right)}^{2}-\frac{\partial Y(cw,\theta )}{\partial cw}\right)]<0 \end{array}$$

Therefore we can have ∂S(cw,θ)/∂cw<0 and $\partial^{2}S\left(cw,\theta \right)/\partial cw^{2}>\;0$. We can conclude that ∂S(cw,θ)/∂cw monotonously approaches 0 with respect to cw. Therefore, for any $cw>cw_{ref}$ we can have

(35) $$max\{|\frac{\partial S(t,\theta )}{\partial t}|,t\in [cw_{ref},cw]\}\;=\;|\frac{\partial S(cw_{ref},\theta )}{\partial cw_{ref}}|$$

According to Equation (4), we can calculate the upper bound of N(cw) in Equation (36) for any $cw>cw_{ref}$.

(36) $$\left|N\left(cw\right)\right|\leq \left(cw-cw_{ref}\right)\cdot \left|\frac{\partial S(cw_{ref},\theta )}{\partial cw_{ref}}\right|$$

From the definition of Λ(cw,θ), we can see that $L_{data}$ and $T_{S}$ are independent of S(cw,θ). Therefore, we set $L_{data}=1KB$ for simplicity in the next section.

#### 2.2.5. Model Evaluation

According to the above analysis, the upper bound of |N(cw)| is controlled by Equations (16), (24), (29) and (36). We set the maximum contention window size $cw_{max}=10000$ in this paper since this size is large enough even for super dense networks. According to the monotone property of $P_{I}\left(cw,\theta \right)$, $P_{S}\left(cw,\theta \right)$, $P_{C}\left(cw,\theta \right)$ and S(cw,θ), we have:

(37) $$|N\left(cw\right)|\leq |N\left(cw_{max}\right)|$$

We can calculate the upper bound of |N(cw)| for $cw\in [cw_{ref},cw_{max}]$ through Equations (16), (24), (29) and (36) respectively.

In order to improve the estimation accuracy, we introduce sub-cases by $cw=cw_{i},i=0,1,2,...,I_{n}$ ($I_{n}$ is a counter of the number of target sub-cases), and we have $cw_{ref}=cw_{0}<cw_{1}<cw_{2}<\cdot \cdot \cdot <cw_{I_{n}}<cw_{max}$. Then $|N\left(cw\right)|,cw\in \left[cw_{ref},cw_{max}\right]$ can be calculated according to Equation (38) below,

(38) $$\begin{array}{c} |N\left(cw\right)|\leq |N\left(cw_{max}\right)|\;<\sum_{i=0}^{I_{n}-1}\left(cw_{i+1}-cw_{i}\right)\cdot |\frac{\partial C_{1}\left(cw_{i},\theta \right)}{\partial cw_{i}}|\;+\left(cw_{max}-cw_{I_{n}}\right)\cdot \left|\frac{\partial C_{1}\left(cw_{I_{n}},\theta \right)}{\partial cw_{I_{n}}}\right| \end{array}$$

**Figure 1:**
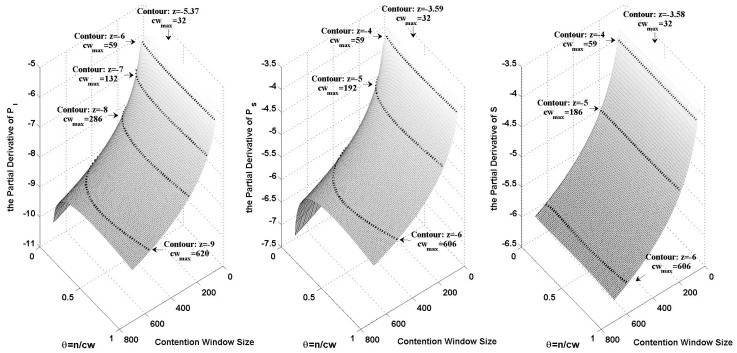
The logarithmic variance of $\partial P_{I}\left(cw,\theta \right)/\partial cw$, $\partial P_{S}\left(cw,\theta \right)/\partial cw$ and ∂S(cw,θ)/∂cw, when cw changes from 32 to 700 and *θ* changes from 0 to 1.

Figure 1 shows the logarithmic variance of $\partial P_{I}\left(cw,\theta \right)/\partial cw$, $\partial P_{S}\left(cw,\theta \right)/\partial cw$ and ∂S(cw,θ)/∂cw when cw changes from 32 to 700 and *θ* changes from 0 to 1. In the figure, we plotted the contours of different orders of magnitude. The maximum contention window size on each contour has been marked.

Based on both Figure 1 and Equation (38), we can calculate |N(cw)|. We take an example where the channel contention level is indicated by the probability of an idle slot. By letting $[cw_{0}=32$, $cw_{1}=59,cw_{2}=132,cw_{3}=286,cw_{4}=620]$, we can get $|N\left(cw\right)|<2.48\times 10^{-4}$ from Equation (38) as shown in Figure 1. Table 1 shows |N(cw)| in the case where we have $cw_{ref}=32,64,128,256$. As for the throughput, |N(cw)| is normalized by being divided by $S(cw_{ref},\theta_{opt})$ which is the optimal throughput in this case.

**Table 1:** The certification results of |N(cw)| in the cases where channel contention level is indicated by different channel parameters.

	${\mathrm{cw}}_{\mathrm{ref}}=32$	${\mathrm{cw}}_{\mathrm{ref}}=64$	${\mathrm{cw}}_{\mathrm{ref}}=128$	${\mathrm{cw}}_{\mathrm{ref}}=256$
$C_{1}=P_{i}$	$2.48\times 10^{-4}$	$9.20\times 10^{-5}$	$2.88\times 10^{-5}$	$1.32\times 10^{-5}$
$C_{1}=P_{s}$	$2.77\times 10^{-2}$	$1.80\times 10^{-2}$	$7.70\times 10^{-3}$	$4.10\times 10^{-3}$
$C_{1}=P_{c}$	$2.75\times 10^{-2}$	$1.79\times 10^{-2}$	$7.70\times 10^{-3}$	$4.10\times 10^{-3}$
$C_{1}=S$	3.08%	2.06%	0.87%	0.46%

## 3. Adaptive Multi-Level Contention Window Tuning Algorithm

In the previous sections, we introduced normalized contention analysis model through which every possible channel state is effectively mapped onto a reference case. In this section, we propose a multi-level contention window tuning scheme with contention window tuning rate adaptively adjusted based on the channel contention information.

### 3.1. Basic Contention Window Tuning under Normalized Model

As shown in Table 1, |N(cw)| in $P_{I}\left(cw_{ref},\theta \right)$ is smaller than that in $P_{s}\left(cw_{ref},\theta \right)$ and $P_{c}\left(cw_{ref},\theta \right)$ in the normalized model. Therefore, we estimate the probability of idle slot state to control the channel contention in our proposed algorithm. As the estimation module with the sample size of hundreds can only effectively distinguish the deviations which are larger than $10^{-3}$, $\left|N\left(cw\right)\right|\leq 2.48\times 10^{-4}$ can hardly be detected. Therefore, $cw_{ref}=32$ is applied in the following analysis.

**Figure 2:**
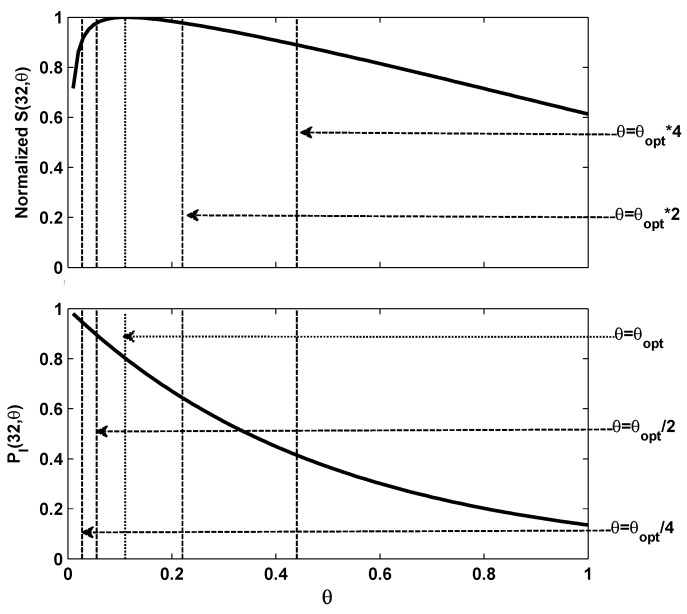
The $P_{I}\left(32,\theta \right)$ and the normalized S(32,θ) vary with different *θ*.

We make $CW_{MAX}=10,000$ as the maximum contention window size and $CW_{MIN}=32$ as the minimum contention window size. The probability of the idle slot state of the channel at all different contention level can be calculated through $P_{I}\left(cw_{ref},\theta \right)=P_{I}\left(cw_{ref},n/cw\right)$. Figure 2 shows the relationship between graph of $P_{I}\left(cw_{ref},\theta \right)$ and the normalized $S(cw_{ref},\theta )$ which is obtained by $S\left(cw_{ref},\theta \right)/\max\left\{S\left(cw_{ref},\theta \right)\right\}$. As shown by the figure, a one-one mapping can be obtained between the probability of an idle slot in channel and the normalized throughput.

From the figure above, we can easily get $\theta_{opt}$ corresponding to the optimal overall throughput. The probability of the idle slot state can be calculated using $P_{I}\left(cw_{ref},\theta_{opt}\right)$. In order to achieve maximum throughput, *θ* should stay at its optimal value, which can be fully indicated by $P_{I}\left(cw_{ref},\theta \right)$. We denote $\hat{P_{I}}$ as the estimated $P_{I}\left(cw_{ref},\theta \right)$. If we detect $\hat{P_{i}}>P_{I}\left(cw_{ref},\theta_{opt}\right)$, we know a smaller cw is required to pull the *θ* back to $\theta_{opt}$, and likewise if we detect $\hat{P_{I}}<P_{I}\left(cw_{ref},\theta_{opt}\right)$, then cw should be increased.

### 3.2. Adaptive Contention Window Tuning

As we know, the number of contending nodes may vary from tens to hundreds within a short time period depending on if nodes have data for transmission. The dynamics of number of contending nodes will deviate $P_{I}\left(cw_{ref},\theta \right)$ from its optimal value. The contention window tuning rate depends on the specific number of contending nodes and how fast it changes. If the number of contending nodes changes slowly, we have enough time to tune the contention window to match the current number of contending nodes. If a large number of contending nodes join the contention, a much faster tuning process is required to make sure the contention window reaches the optimal value on time. Unfortunately, these two situations can hardly be solved using one single contention window tuning rate because a more refined tuning requires a smaller tuning rate, while a faster tuning needs a larger turning rate. In order to solve the problem above, we propose an adaptive contention window tuning strategy in which the contention window tuning rate can be set dynamically according to the real-time channel contention level.

#### 3.2.1. Algorithm Design

In the normalized model, a sudden increase of contending nodes can be represented by the increase of *θ* through the mapping of θ=n/cw. Based on the one-to-one mapping in Figure 2, we can see that the probability of an idle slot state is exactly determined by *θ*. It means that the amplitude of changing in the number of contending nodes can be indicated by the variance of $P_{I}\left(cw_{ref},\theta \right)$. Therefore, contention window tuning rates can be determined in real-time adaptively based on $\hat{P_{I}}$.

In Figure 2, we can see the relationship between $P_{I}\left(cw_{ref},\theta \right)$ and the corresponding overall throughput by dotted lines in the cases where *θ* suddenly increases to 2 and 4 times of $\theta_{opt}$, and then decreases to half and quarter of $\theta_{opt}$. Just the same as the basic contention window tuning processing during which we believe *θ* has increased when $\hat{P_{I}}$ is smaller than $P_{i}\left(cw_{ref},\theta_{opt}\right)$, and *θ* has at least doubled when the $\hat{P_{I}}$ is smaller than $P_{I}\left(cw_{ref},2\cdot \theta_{opt}\right)$. Therefore, the contention window can be increased again to make sure *θ* is approaching $\theta_{opt}$ faster. Similarly the contention window size should be increased again if $\hat{P_{I}}<P_{I}\left(cw_{ref},4\cdot \theta_{opt}\right)$ happens. The similar rules will be applied so that if $\hat{P_{I}}>P_{I}\left(cw_{ref},\theta_{opt}/2\right)$ or $\hat{P_{I}}>P_{I}\left(cw_{ref},\theta_{opt}/4\right)$ happens, the contention window should be decreased by half each time.

Based on the discussions above, the *M*-Level contention window tuning algorithm with a minimum tuning rate of *γ* is proposed in Algorithm 1. In this algorithm, the decisions on how to change contention window size and tuning rate are made based on a pair of vectors: vector_inc[M] and vector_dec[M]. Each vector divides the contention channel into M different contention levels according to its *M* ordered elements using *M* thresholds of $P_{I}\left(cw_{ref},\theta \right)$. As shown in Algorithm 1, the ordered thresholds of $P_{I}\left(cw_{ref},\theta \right)$ are defined as $P_{I}\left(cw_{ref},\theta_{opt}\cdot \gamma^{i}\right),i\in \left[0,M-1\right]$ and $P_{I}\left(cw_{ref},\theta_{opt}/\gamma^{i}\right),i\in \left[0,M-1\right]$, which are pre-defined during the initialization period. A node running *M*-Level contention window tuning algorithm will compare the $\hat{P_{I}}$ with the elements of the vector. Once $\hat{P_{I}}$ is smaller than one of the elements in vector_inc[M], the contention window would be multiplied by *γ*. Likewise, if the $\hat{P_{I}}$ is larger than one of the elements in vector_dec[M], the contention window would be divided by *γ*. Based on the above, the update of *M* contention window tuning rate can be specified as $\left[\gamma ,\gamma^{2},\ldots ,\gamma^{M}\right]$ according to different channel contention levels.

**Algorithm 1** A *M*-Level contention window tuning algorithm with a minimum tuning rate of *γ*.**Initialization**
1:unsigned slot_cnt=0, idle_slot_cnt=0, *k*;2:double cw=CW_MIN, vector_inc[M], vector_dec[M];3:**for** k=0;k<M;k++ **do**4: $vector\_inc\left[k\right]=P_{I}\left(32,\theta \cdot \gamma^{k}\right)$;5: $vector\_dec\left[k\right]=P_{I}\left(32,\theta /\gamma^{k}\right)$;6:**end for**
**Algorithm Body**
1:**while 1 do**2: **if** detecting an idle slot **then**3:  slot_cnt++;4:  **if** the slot is over with idle **then**5:   idle_slot_cnt++;6:  **end if**7: **end if**8:**end while**9:**while 1 do**10: **if** before generating a new backoff period **then**11:  **if** slot_cnt−idle_slot_cnt≥5 **then**12:   **for** k=0;k<M;k++ **do**13:    **if** idle_slot_cnt/slot_cnt<vector_inc[k] **then**14:     cw=cw*γ;15:    **end if**16:    **if** idle_slot_cnt/slot_cnt>vector_dec[k] **then**17:     cw=cw/γ;18:    **end if**19:   **end for**20:   slot_cnt=0, idle_slot_cnt=0;21:  **end if**22: **end if**23:**end while**


#### 3.2.2. The Selection of *M*

Through our proposed *M*-level contention window tuning algorithm, the contention window tuning accuracy can be improved by adopting a small *γ*, and a high tuning rate can also be achieved by applying large *M*. While the *γ* can be selected according to the need, the range of *M* is very limited. It is because the algorithm is based on the assumption that the thresholds including vector_inc[k], k∈[0,M-1] and vector_dec[k],k∈[0,M-1] can be represented by $\hat{P_{I}}$. When the thresholds are very close to each other and the sample size which is used to calculate $\hat{P_{I}}$ is very small, the difference among thresholds is too small to matter. For example, when $\hat{P_{I}}$ is calculated based on 10 samples of the channel, the thresholds with the value of 0.71 and 0.74 could hardly be identified because the resolution of $\hat{P_{I}}$ is around 0.1 which is decided by the samples.

When selecting *M*, we should ensure the difference between neighboring thresholds is large enough to be represented by the $\hat{P_{I}}$. As shown in Algorithm 1, $\hat{P_{I}}$ is calculated based on the consecutive idle slots. We firstly start with a special case where idle_slot_cnt=slot_cnt. In this case, $\hat{P_{I}}=1$ which means the channel is always be busy which is obviously it impossible. Therefore, in order to make $\hat{P_{I}}$ valid, $\hat{P_{I}}$ should not be updated unless a busy slot is detected.

We consider another case where $\hat{P_{I}}$ is used to update the contention window after each busy slot. In this case, the sample size right before calculating $\hat{P_{I}}$ can be interpreted as the estimation of the average number of consecutive idle slots between two busy slots. As the probability of an idle channel in a certain slot is a 0-1 distribution with probability of $P_{I}\left(cw_{ref},\theta \right)$, the average number of consecutive idle slots between two busy slots can be expressed as $1/P_{I}\left(cw_{ref},\theta \right)$. Therefore, we can calculate the sample size of $\hat{P_{I}}$ through Equation (39).

(39) $$SS\left(\theta \right)=\left[\frac{1}{P_{I}\left(cw_{ref},\theta \right)}\right]$$

The sample size can be obtained based on *θ*, and the sample sizes of $\hat{P_{I}}$ for all the thresholds in vector_inc and vector_dec can be calculated using SS(vector_inc[k]),k∈[0,M-1] and SS(vector_dec[k]),k∈[0,M-1]. If Equation (40) holds, $P_{I}\left(cw_{ref},vector\_inc\left[k\right]\right),k\in \left[0,M-1\right]$ and $P_{I}\left(cw_{ref},vector\_dec\left[k\right]\right),k\in \left[0,M-1\right]$ can be totally differentiated because the numbers of consecutive idle slots between two busy ones for different thresholds are quite different, and these differences can be effectively indicated by $\hat{P_{I}}$ after each busy slot. Obviously a maximal *M* can be derived from Equations (39) and (40).

(40) $$\left\{\begin{array}{c} \left|SS(vector\_inc[u])-SS(vector\_inc[v])|\geq 1,u,v\in [0,M-1\right]\\ \left|SS(vector\_dec[u])-SS(vector\_dec[v])|\geq 1,u,v\in [0,M-1\right] \end{array}\right.$$

In order to get larger *M*, we can limit the minimum number of busy slots before calculating $\hat{P_{I}}$ to 5 in this paper, and then we can have $SS\left(\theta \right)=[5/P_{i}\left(32,\theta \right)]$. According to the requirement in Equation (40), Table 2 lists the maximal *M* for different *γ*.

**Table 2:** The maximal *M* and corresponding equivalent maximal contention window tuning rate ($\gamma_{max}$) in *M*-level contention window tuning algorithm for different *γ* ranging from 1.2 to 2.0.

***γ***	1.2	1.3	1.4	1.5	1.6	1.7	1.8	1.9	2.0
M	10	9	9	7	7	6	6	5	5
$\gamma_{\max}$	5.16	8.16	14.76	11.39	16.78	14.20	18.90	13.03	16.00

The contention window size will be updated if and only if the number of busy slots $\hat{P_{I}}$ is larger than 5 in our case. As the contention window tuning will only be triggered when a node is about to access the channel, the number of busy slots represented by $\hat{P_{I}}$ will be always larger than 5. This can sufficiently ensure the differentiation of thresholds in *M*-level contention window tuning algorithm. Now the *M*-level contention window tuning algorithm with parameters of *M* and *γ* as inputs can be fully constructed. In next section, we will focus on the performance evaluation of our proposed algorithm.

## 4. Performance Evaluation of *M*-level Contention Window Tuning Algorithm

In this section, we study the performance of the proposed algorithm using OMNET++ simulator [29]. Some of the PHY and MAC layer parameters are listed in Table 3 based on the IEEE 802.11b standard [9]. The reason for studying the performance based on IEEE 802.11b network is that IEEE 802.11b supports longer distance than that of IEEE 802.15.4, so that the number of contending nodes in IEEE 802.11 networks can be much larger than that in IEEE 802.15.4 networks. Since the backoff processing is universal in all the IEEE 802.11x and IEEE 802.15.4 standards, the proposed algorithm and its performance study work for all the backoff based medium access schemes in IEEE 802.11x and 802.15.4 networks.

**Table 3:** PHY layer and MAC layer parameters used in simulation.

Parameters	Value	Parameters	Value
Channel Bit Rate	11 Mbps	Payload Length	1 KB
Slot Time(ST)	20 μs	MAC Header	224 bit
SIFS	10 μs	RTS	160 bit
DIFS	50 μs	CTS	112 bit
PHY Header	192 μs	ACK	112 bit

We apply two different types of networks in terms of node density: sparse networks where the number of contending nodes is from 4 to 20, and the super dense networks where the number of contending nodes can be up to 400. We firstly study the case when the number of contending nodes remains static during the whole simulation period, and then consider the dynamic case where the number of contending nodes changes as time goes.

### 4.1. Simulations in Sparse Networks

In this section, the throughput and fairness of the proposed algorithm are studied in sparse networks where the number of contending nodes varies from 4 to 20. 8 groups of simulations are conducted with different parameters for algorithms including BEB [6] and the idle sense algorithm [23], which is a classical and efficient estimation-based algorithm. We choose different *γ* and *M* for our proposed algorithm.

**Figure 3:**
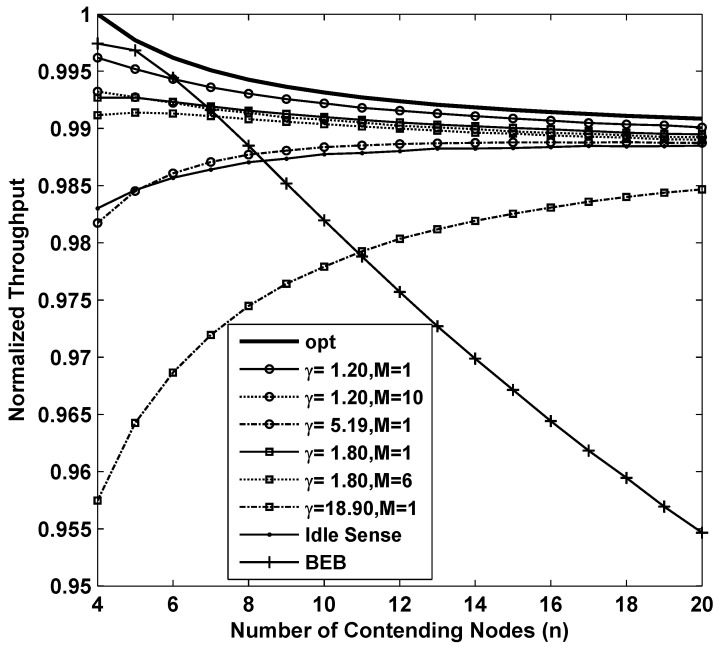
The normalized throughput performance of *M*-level contention window tuning algorithm with different *M* and *γ*, idle sense algorithm and BEB for sparse deployed networks.

Figure 3 shows the results of the throughput while applying different backoff algorithms. In the figure, the curve named opt is the result of $S(cw,\theta_{opt})$ which is the optimum throughput that CSMA/CA based networks can achieve theoretically. From the figure we can see that BEB is efficient when the number of contending nodes is very small. However when number of contending nodes increases, the throughput decreases rapidly. This is because BEB algorithm can not estimate the channel contention accurately enough to fully utilize the channel when the channel contention becomes intense. The throughput of idle sense algorithm decreases when the number of contending nodes is small. This is because a smaller number of contending nodes has a shorter backoff time, so that a small number of samples could be obtained during the backoff period. The limit on the number of samples will lead to inaccurate estimates. The inaccurate estimates together with the unbalanced contention window tuning rate in idle state normally will introduce errors to the contention window size. This problem is effectively solved in our proposed algorithm. Two simulations of our proposed algorithm with parameters of (γ=1.2,M=10) and (γ=1.8,M=6) are presented in the figure. In order to evaluate their performance, we compare with the performance of the proposed algorithm with settings of (γ=1.2,M=1) and (γ=1.8,M=1). We also found that the throughput with (γ=1.2,M=10) and (γ=1.8,M=6) is much lower than that of other cases. This is mainly because inaccurate estimates result in even larger contention window deviation from the optimal size in *M*-level tuning scheme. Nevertheless, the overall throughput of our proposed algorithm is very close to the optimal value with a loss of no more than 1% for all the settings. We also studied the throughput of with $\left(\gamma =1.2^{10}=5.19,M=1\right)$ and $\left(\gamma =1.8^{6}=18.9,M=1\right)$. From the figure, we can see that larger contention window tuning rate decreases the throughput in sparse networks because tuning of contention window size happens very frequently even when the number of contending nodes changes slightly. Therefore, we can conclude that in order to achieve high throughput performance in sparse networks, small contention window tuning rate is necessary.

**Figure 4:**
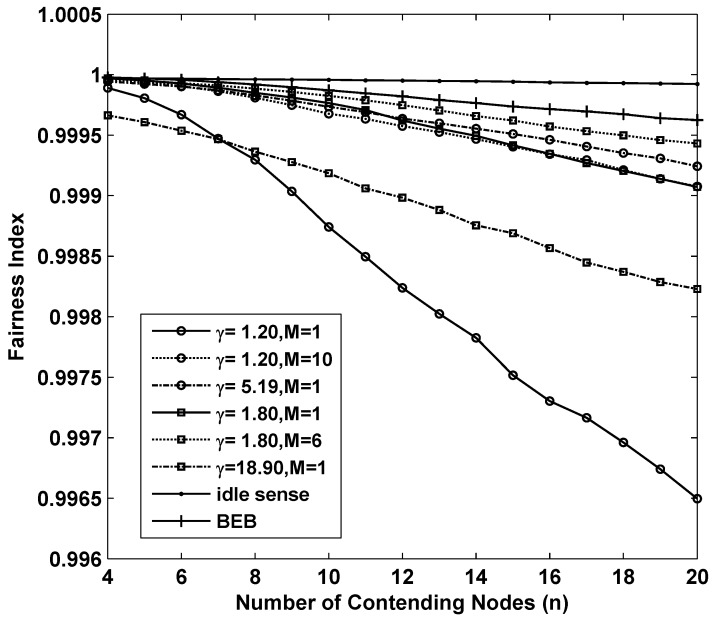
The fairness performance of *M*-level contention window tuning algorithm with different *M* and *γ*, idle sense algorithm and BEB for sparse deployed networks.

In estimate-based backoff algorithms, all nodes try to improve the channel utilization rate to approach the optimal rate. However, during the process, the contention window size on each contending node is different when network topology changes or estimate error occurs, and this may remain for quite a long time. Therefore, the fairness among nodes is another key factor of estimate-based backoff algorithms. In this paper, we apply the fairness index concept defined by Jain [30] to evaluate the fairness of each algorithm. The fairness index is defined as:

(41) $$\begin{array}{c} FI=\frac{{\left(\sum_{i}TH_{i}\right)}^{2}}{n\cdot \sum_{i}TH_{i}^{2}} \end{array}$$

in which *n* is the number of contending nodes, and $TH_{i}$ is the throughput of node *i*. Based on the Cauchy-Schwartz inequality, we obtain FI≤1, and the equality holds if and only if all $TH_{i}$ are equal. Thus, the more FI approaches to 1, the better fairness the algorithm can achieve. Figure 4 shows the fairness of the 8 sets of simulations we analyzed above. Fortunately all of them achieved remarkable fairness which is above 0.995 where the number of contending nodes ranges from 4 to 20.

### 4.2. Performance in Super Dense Networks

In this section, the throughput and fairness are evaluated in super dense networks where the number of contending nodes varies from 10 to 400. Because smaller contention window tuning rate is required in sparse deployed networks and the throughput of BEB declines severely when the number of contending nodes reaches 20, in this section we focus on the performance of idle sense algorithm and our proposed algorithm with parameters of (γ=1.2,M=10), (γ=1.8,M=6), (γ=1.2,M=1) and (γ=1.8,M=1).

**Figure 5:**
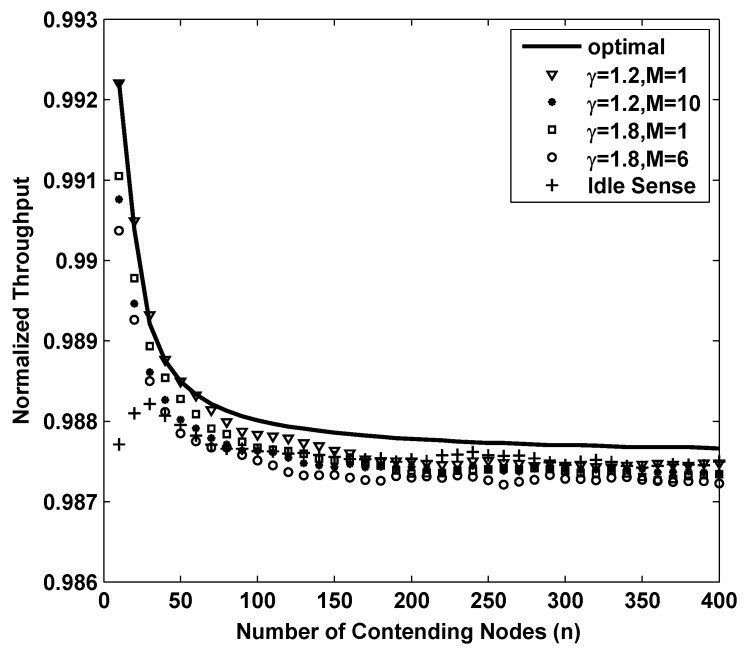
The normalized throughput performance of *M*-level contention window tuning algorithm with different *M* and *γ* and idle sense algorithm for super dense deployed networks.

Figure 5 shows the simulation results of network throughput of different backoff algorithms. The curve named opt in the figure is the result of theoretical optimal throughput calculated by $S(cw,\theta_{opt})$. From the figure we can see that the throughput of simulation always oscillates around a certain level. It is reasonable because the contention window size of each node may be enumerated, and it is only optimal for some contending nodes. In other cases, the throughput slightly decreases because of the deviation of contention window size from their optimal size. The throughput with (γ=1.2,M=1), (γ=1.8,M=1), and that of the idle sense algorithm are all very close to the optimal value. The throughput of *M*-level contention window tuning is almost equivalent to that of 1-level tuning case and that of idle sense algorithm, and the overall differentiation is no more than 0.5%.

**Figure 6:**
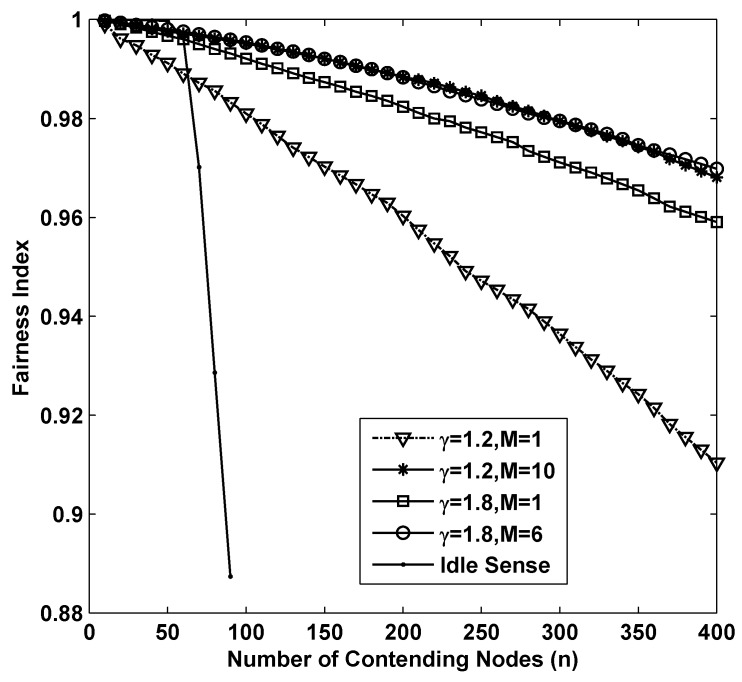
The fairness performance of *M*-level contention window tuning algorithm with different *M* and *γ* and idle sense algorithm for super dense deployed networks.

The fairness of different algorithms in super dense deployed networks is shown in Figure 6. From the figure we can see that as the number of contending nodes increases, there is a significant decline of the fairness in all the simulations. In sparse networks, smaller contention tuning rate improves the throughput, however it has negative impact on the fairness. This is because differential contention window sizes with different contending nodes are much easier to be increased during a substantial change of contention window size through small amplitude adjustments. Therefore, the fairness of idle sense algorithm decreases rapidly after the number of contending nodes is larger than 50. However, the superiority of M-level contention window tuning algorithm is fully demonstrated in the simulations. The fairness indexes for (γ=1.2,M=10), (γ=1.8,M=6) are larger than 0.97 even when the number of contending nodes increases to 400.

### 4.3. Simulations in Very Dynamic Super Dense Networks

According to the definition of *θ* in Section 2.1, a sudden change of the number of contending nodes will make *θ* deviates from its optimal value. As shown in Figure 2, throughput declines with respect to such deviations. Backoff algorithms detect such changes and try to make *θ* back to optimal by tuning the contention window. Thus, the contention window tuning efficiency can be represented by the time spent on.

**Figure 7:**
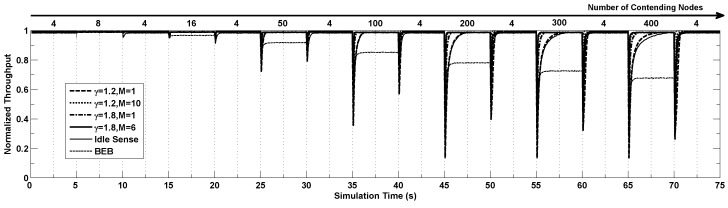
The normalized throughput performance of *M*-level contention window tuning algorithm with different *M* and *γ*, idle sense algorithm and BEB when the number of contention nodes changes.

In this section we study the performance of our proposed algorithm in settings where the channel contention changes frequently as the number of nodes contending the channel changes dramatically. In the simulations, the number of contending nodes changes following steps of (4, 8, 4, 15, 4, 40, 4, 100, 4, 200, 4, 300, 4, 400 and 4) and each step lasts for 5 seconds. Figure 7 shows the dynamics of throughput of different algorithms running the simulation above. From the figure we can see that the throughput becomes extremely low when the number of contending nodes changes suddenly, and the throughput gradually increases to the optimal value after a certain period of time with the help of the proposed algorithm. When the number of contending nodes changes in small scale, all the algorithms have similar performance. However when the number of contending nodes changes tremendously, the throughput of BEB decrease very much. The idle sense algorithm has the slowest contention window tuning speed because of its small contention window tuning rate as shown by the Figure. Similar situation appears in our proposed algorithm with (γ=1.2,M=1). In the situation where the number of contending nodes changes from 4 to 400, their adaptation time is more than 3 s. By applying the larger γ=1.8, the adaptation time is effectively reduced to around 2s. Our proposed M-level tuning algorithm can reduce the adaptation time dramatically since a much larger contention window tuning rate can be applied when the channel contention becomes much more intensive according to our algorithm. From the figure we can see that for the situations where n≤400, our proposed algorithm with parameters of (γ=1.2,M=10) and (γ=1.8,M=6) has a very small adaptation time smaller than 0.5 s.

## 5. Conclusions

In this paper, an adaptive and efficient channel access method for super dense wireless sensor networks was proposed. By constructing and verifying a normalized channel contention model, we mapped the diverse channel parameters onto reference cases. Our proposed model significantly reduces the complexity of channel contention analysis. Based on this normalized model, a multi-level contention window tuning algorithm was proposed. By estimating the channel contention level based on the deviation degree of the channel parameters from its optimal value, the contention window tuning rate can be set dynamically and adaptively according to the number of contending nodes to accelerate the contention window adaptation process.

To evaluate the performance of the proposed algorithm, we performed comprehensive simulations in sparse networks, super dense networks and dynamic networks. Simulation results show that our proposed algorithm can provide stable throughput and fairness performance close to the theoretical throughput bound in networks with different node densities and dynamics. Our algorithm can also achieve high contention window tuning speed in dynamic networks where the number of active nodes changes rapidly.
